# Synthesis of medium and large phostams, phostones, and phostines

**DOI:** 10.3762/bjoc.19.50

**Published:** 2023-05-15

**Authors:** Jiaxi Xu

**Affiliations:** 1 State Key Laboratory of Chemical Resource Engineering, Department of Organic Chemistry, College of Chemistry, Beijing University of Chemical Technology, Beijing 100029, People’s Republic of China; 2 College of Sciences, Henan Agricultural University, Zhengzhou 450002, P. R. China

**Keywords:** azaphosphaheterocycle, oxaphosphaheterocycle, phostam, phostine, phostone

## Abstract

Phostams, phostones, and phostines are a series of 1,2-azaphosphaheterocycle and 1,2-oxaphosphaheterocycle 2-oxide derivatives. They are phosphorus analogues of lactams and lactones and important biologically active compounds. The strategies for the synthesis of medium and large phostams, phostones, and phostines are summarized. They include cyclizations and annulations. Cyclizations achieve ring construction through the formations of C–C, C–O, P–C, and P–O bonds in the rings, while annulations build the rings via [5 + 2], [6 + 1], and [7 + 1] fashions with the stepwise formation of two ring bonds. This review includes the recent syntheses of seven to fourteen-membered phostam, phostone, and phostine derivatives.

## Introduction

**Figure 1:**
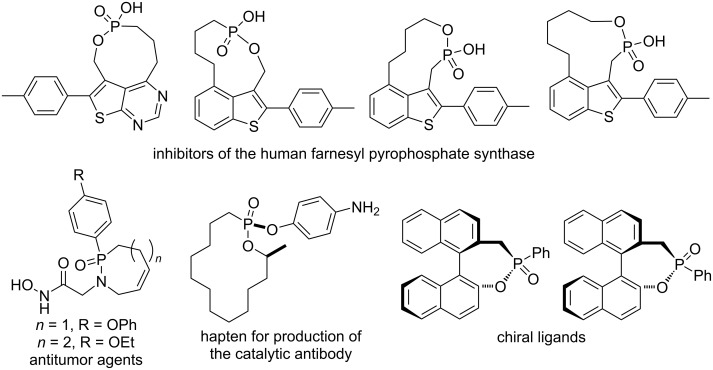
Biologically active agents and chiral ligands containing medium and large phostams, phostones, and phostines.

Phostams, phostones, and phostines are a class of important phosphorus-containing heterocyclic compounds [1–2] and significant agrochemicals [3], medicinal agents [4], and organic materials [5]. They are also organic synthetic intermediates and building blocks [6–7]. To date, only limited four-membered β-phostams [8–9], β-phostones and β-phostines [10] have been prepared, while numerous five-membered γ-phostams [11–12], γ-phostones and γ-phostines [13], and six-membered δ-phostams [14–16], δ-phostones [17–18] and δ-phostines [17,19–20] have been synthesized via various synthetic strategies. In comparison with common ring size phostam, phostone, and phostine derivatives, less attention has been paid to the synthesis of medium and large phostam, phostone, and phostine derivatives. They are also biologically active compounds, such as inhibitors of the human farnesyl pyrophosphate synthase [21–22], antitumor agents [23], and hapten for the production of the catalytic antibody [24]. They are also potential chiral ligands in asymmetric catalysis [25] (Figure 1).

**Figure 2:**
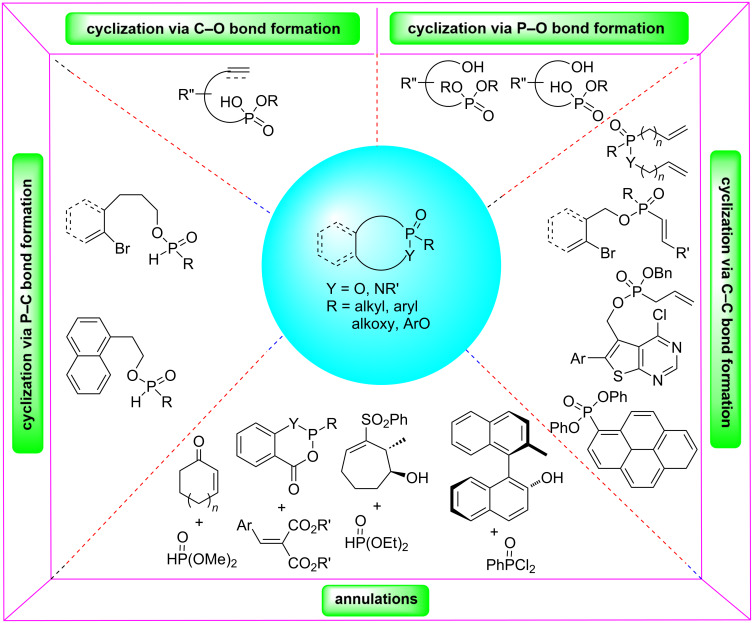
Synthetic strategies for the preparation of medium and large phostams, phostones, and phostines.

Cyclizations and annulations are two major strategies for the synthesis of medium and large phostam, phostone, and phostine derivatives. The cyclizations have been applied in the construction of C–C, C–O, P–C, and P–O bonds in the rings, while annulations are composed of [5 + 2], [6 + 1], and [7 + 1] fashions for the formation of the rings (Figure 2). This review includes the synthesis of seven to fourteen-membered phostam, phostone, and phostine derivatives. Aboundant methods have been developed for the synthesis of seven-membered phostone and phostine derivatives.

## Review

### 1 Synthesis via cyclizations

Cyclizations are major strategies for the construction of medium and large phostams, phostones, and phostines via C–C, C–O, P–C, and P–O bond formations, respectively. These strategies can be applied for the synthesis of seven to fourteen-membered phostam, phostone, and phostine derivatives.

#### 1.1 Synthesis via C–C bond formation

Most medium and large phostams, phostones, and phostines were prepared via C–C bond formation, especially via ring-closing metathesis (RCM).

**1.1.1 Synthesis via C–C bond formation through RCM reaction:** Ring-closing metathesis (RCM) is an efficient strategy for the construction of common to large cyclic compounds via the formation of a C=C bond [26], which can be further reduced to a C–C bond.

**Scheme 1:**
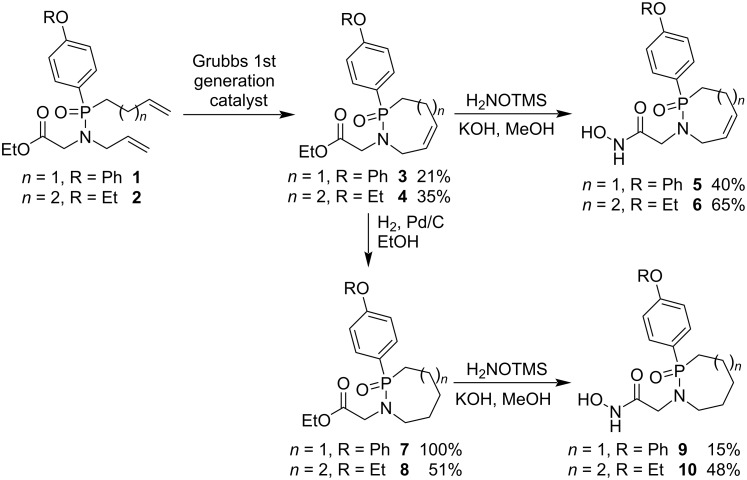
Synthesis of 1,2-azaphosphepine 2-oxide, 1,2-azaphosphocine 2-oxide, 1,2-azaphosphepane 2-oxide, and 1,2-azaphosphocane 2-oxide from ethyl *N*-allyl-*N*-(alkenyl(4-phenoxyphenyl)phosphoryl)glycinates.

To prepare phostam-derived antitumor agents, ethyl *N*-allyl-*N*-(but-3-en-1-yl(4-phenoxyphenyl)phosphoryl)glycinate (**1**) and ethyl *N*-allyl-*N*-((4-ethoxyphenyl)(pent-4-en-1-yl)phosphoryl)glycinate (**2**) were prepared and cyclized into ethyl 2-(2-oxido-2-(4-phenoxyphenyl)-3,4,7-trihydro-1,2-azaphosphepin-1-yl)acetate (**3**) and ethyl 2-(2-(4-ethoxyphenyl)-2-oxido-3,5,8-trihydro-1,2-azaphosphocin-1(4*H*)-yl)acetate (**4**), respectively, in the presence of the Grubbs first generation catalyst via ring closing metathesis. The products **3** and **4** were further transformed to antitumor agents **5**, **6**, **9** and **10** through aminolysis with *O*-TMS hydroxylamine or hydrogenolysis followed by aminolysis with *O*-TMS hydroxylamine (Scheme 1) [23]. The RCM reaction is a powerful strategy for the synthesis of *P*-stereogenic 3,4,7-trihydro-1,2-azaphosphepine 2-oxide and 1,3,4,5,8-pentahydro-1,2-azaphosphocine 2-oxide derivatives, unsaturated seven- and eight-membered phostams, which can be further reduced to saturated 1,2-azaphosphepane 2-oxide and 1,2-azaphosphocane 2-oxide derivatives. Thus, the strategy is an efficient method for the synthesis of 1,2-azaphosphepine 2-oxides, 1,2-azaphosphocine 2-oxides, 1,2-azaphosphepane 2-oxides, and 1,2-azaphosphocane 2-oxides via C–C bond formation.

All phostams **5**, **6**, **9** and **10** are efficient inhibitors of matrix metalloproteinases MMP-1, MMP-3, and MMP-9 with IC_50_ values varied from 5.0 nM to 10.0 μM. They are more efficient against MMP-3 and MMP-9 than against MMP-1. Phostams **5** and **6** show higher inhibition activities than **9** and **10** [23].

**Scheme 2:**
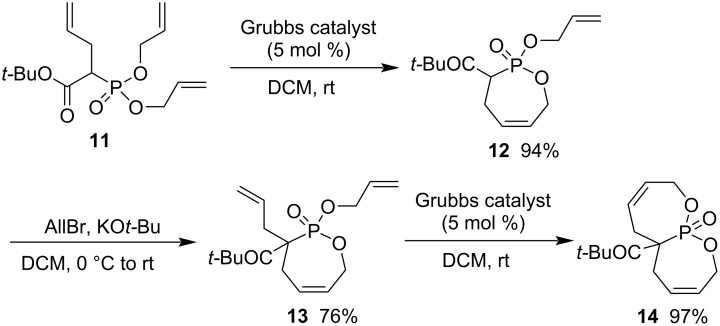
Synthesis of bis[1,2]oxaphosphepine 2-oxide from *tert*-butyl 2-(bis(allyloxy)phosphoryl)pent-4-enoate.

RCM is also an efficient strategy for the synthesis of 1,2-oxaphosphaheterocycle 2-oxides. *tert*-Butyl 2-(bis(allyloxy)phosphoryl)pent-4-enoate (**11**) generated *tert*-butyl 2-(allyloxy)-3,4,7-trihydro-1,2-oxaphosphepine-3-carboxylate 2-oxide (**12**) in excellent 94% yield in the presence of the Grubbs catalyst in DCM. After further allylation with allyl bromide, its allylated product **13** further cyclized into bis(1,2-oxaphosphepine 2-oxide) derivative *tert*-butyl 2,5,5a,6,9-pentahydro-[1,2]oxaphosphepino[2,3-*b*][1,2]oxaphosphepine-5a-carboxylate 11-oxide (**14**) in 97% yield under the same conditions (Scheme 2) [27].

**Scheme 3:**
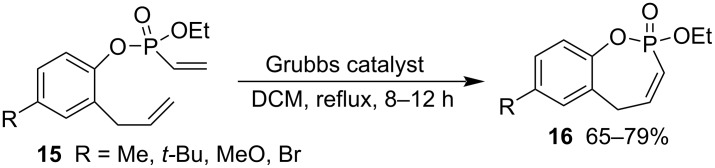
Synthesis of 2-ethoxy-5*H*-benzo[*f*][1,2]oxaphosphepine 2-oxides from 2-allylphenyl ethyl vinylphosphonates.

The Grubbs 1st generation catalyst-promoted ring-closing metathesis of 2-allylphenyl ethyl vinylphosphonates **15** generated 2-ethoxy-5*H*-benzo[*f*][1,2]oxaphosphepine 2-oxides **16** in good yields in refluxing DCM for 8–12 h (Scheme 3) [28].

**Scheme 4:**
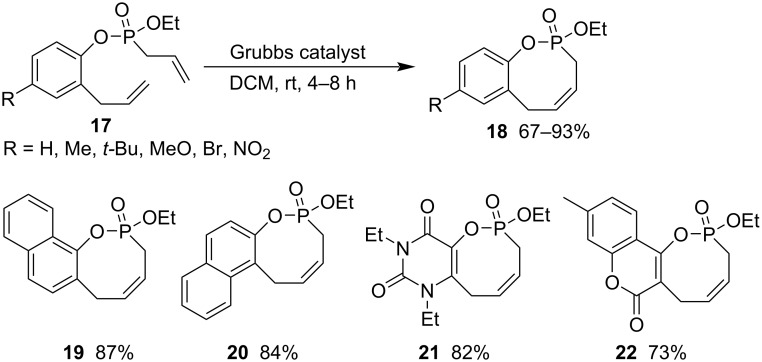
Synthesis of 2-ethoxy-3,6-dihydrobenzo[*g*][1,2]oxaphosphocine 2-oxides from 2-allylphenyl ethyl allylphosphonates.

The ring-closing metathesis of 2-allylphenyl ethyl allylphosphonates **17** gave rise to 2-ethoxy-3,6-dihydrobenzo[*g*][1,2]oxaphosphocine 2-oxides **18** in good to excellent yields in DCM at room temperature for 4–8 h in the presence of the Grubbs 1st generation catalyst. Two naphthylene-fused (**19** and **20**), pyrimidine-2,4(1*H*,3*H*)-dione-fused (**21**), and 2*H*-chromen-2-one-fused (**22**) 1,2-oxaphosphocine 2-oxides were also prepared in good yields following the same procedure (Scheme 4) [28].

**Scheme 5:**
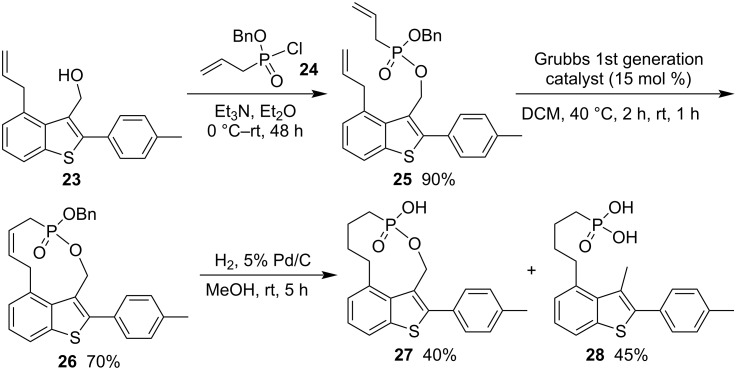
Synthesis of benzothiophene-fused 2-hydroxy-1,2-oxaphosphecane 2-oxide from (4-allyl-2-(4-methylphenyl)benzo[*b*]thiophen-3-yl)methanol and benzyl allylphosphonochlordiate.

To prepare new inhibitors and therapeutical agents of relevant protease enzymes, (4-allyl-2-(4-methylphenyl)benzo[*b*]thiophen-3-yl)methyl benzyl allylphosphonate (**25**) was prepared in 90% yield from (4-allyl-2-(4-methylphenyl)benzo[*b*]thiophen-3-yl)methanol (**23**) and benzyl allylphosphonochlordiate (**24**) in the presence of triethylamine in diethyl ether via phosphonylation. It underwent a RCM reaction under the catalysis of Grubbs first generation catalyst in DCM, affording 2-(4-methylphenyl)benzothiophene-fused 2-(benzyloxy)-3,6,7,8,9,10-hexahydro-1,2-oxaphosphecine 2-oxide **26** in 70% yield. After the Pd-catalyzed hydrogenolysis, it was transformed to both cyclic ten-membered phostone 2-(4-methylphenyl)benzothiophene-fused 2-hydroxy-1,2-oxaphosphecane 2-oxide (**27**) in 40% yield and acyclic (4-(3-methyl-2-(4-methylphenyl)benzo[*b*]thiophen-4-yl)butyl)phosphonic acid (**28**) in 45% yield as a byproduct, which was generated from the Pd-catalyzed arylmethylic cleavage under hydrogenolysis conditions (Scheme 5) [22].

**Scheme 6:**
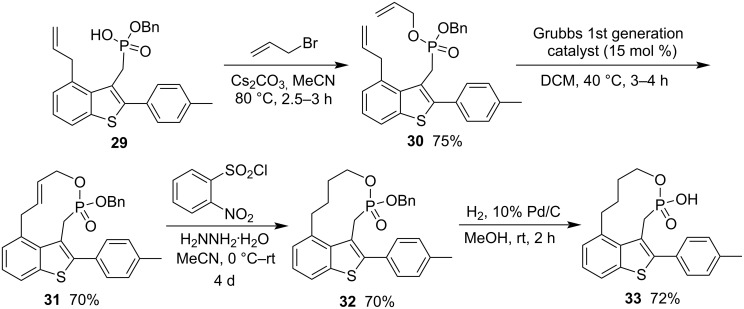
Synthesis of benzothiophene-fused 2-hydroxy-1,2-oxaphosphecane 2-oxide from benzyl hydrogen ((4-allyl-2-(4-methylphenyl)benzo[*b*]thiophen-3-yl)methyl)phosphonate and allyl bromide.

To avoid the formation of the acyclic byproduct, the same research group designed a new inhibitor with a reverse phosphonate bond. Allyl benzyl ((4-allyl-2-(4-methylphenyl)benzo[*b*]thiophen-3-yl)methyl)phosphonate (**30**) was prepared in 75% yield from benzyl hydrogen ((4-allyl-2-(4-methylphenyl)benzo[*b*]thiophen-3-yl)methyl)phosphonate (**29**) and allyl bromide in the presence of Cs_2_CO_3_ in acetonitrile at 80 °C for 2.5–3 h via alkylation. The RCM reaction was performed in the presence of Grubbs first generation catalyst in DCM, affording 2-(4-methylphenyl)benzothiophene-fused 2-(benzyloxy)-3,4,5,6,7,10-hexahydro-1,2-oxaphosphecine 2-oxide **31** in 70% yield. After hydrazine reduction and Pd-catalyzed hydrogenolysis, it was converted into 2-(4-methylphenyl)benzothiophene-fused 2-hydroxy-1,2-oxaphosphecane 2-oxide **33** in 70% and 72% yield for the two steps, respectively, without formation of any acyclic byproduct (Scheme 6) [22].

**Scheme 7:**
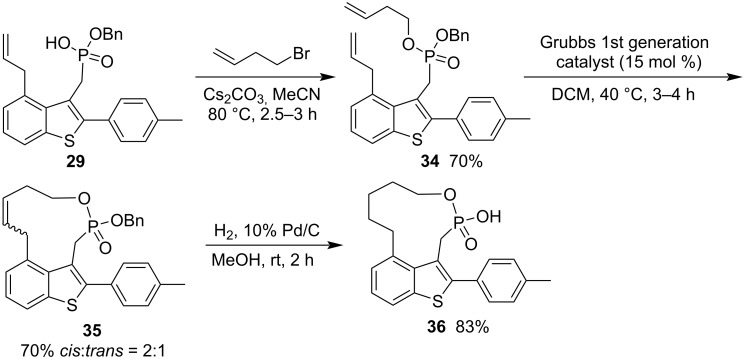
Synthesis of benzothiophene-fused 2-hydroxy-1-oxa-2-phosphacycloundecane 2-oxide from benzyl hydrogen ((4-allyl-2-(4-methylphenyl)benzo[*b*]thiophen-3-yl)methyl)phosphonate and but-3-enyl bromide.

Using the similar way for the synthesis of ten-membered phostones, benzyl but-3-enyl ((4-allyl-2-(4-methylphenyl)benzo[*b*]thiophen-3-yl)methyl)phosphonate (**34**) was prepared in 70% yield from benzyl hydrogen ((4-allyl-2-(4-methylphenyl)benzo[*b*]thiophen-3-yl)methyl)phosphonate (**29**) and but-3-enyl bromide in the presence of Cs_2_CO_3_ in acetonitrile at 80 °C for 2.5–3 h via alkylation. It was cyclized via a RCM reaction with Grubbs 1st generation catalyst in DCM, affording 2-(4-methylphenyl)benzothiophene-fused 2-(benzyloxy)-1-oxa-2-phosphacycloundec-8-ene 2-oxide **35** in 70% yield with a ratio of 2:1 for the generated *cis*- and *trans*-double bonds. After Pd-catalyzed hydrogenolysis, it was converted into 2-(4-methylphenyl)benzothiophene-fused 2-hydroxy-1-oxa-2-phosphacycloundecane 2-oxide **36** in 83% yield (Scheme 7) [22].

**Scheme 8:**
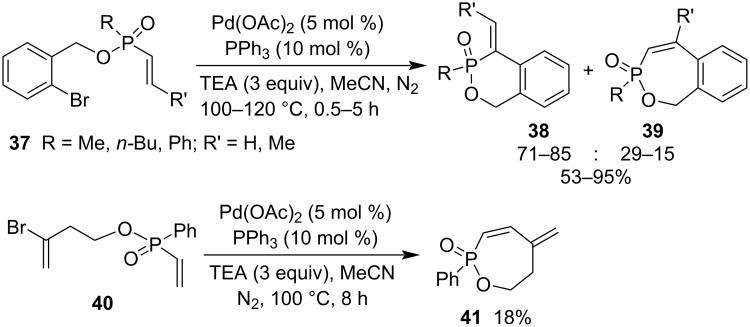
Synthesis of 5,6,7-trihydro-1,2-oxaphosphepine 2-oxide and its benzo derivatives from 3-bromobut-3-en-1-yl ethenyl(phenyl)phosphinate and 2-bromophenylmethyl alk-1-enylphosphinates.

**1.1.2 Synthesis via other C–C bond formations:** The palladium-catalyzed intramolecular Heck arylation of 2-bromophenylmethyl alk-1-enylphosphinates **37** provides access to 4-alkylidene-1,4-dihydrobenzo[*d*][1,2]oxaphosphinine 3-oxides **38** and 1*H*-benzo[*e*][1,2]oxaphosphepine 3-oxides **39** as side-products in moderate to excellent total yields of 53–95% in the presence of triethylamine in dry acetonitrile at 100–120 °C. However, 3-bromobut-3-en-1-yl ethenyl(phenyl)phosphinate (**40**) chemospecifically generated 5-methylene-2-phenyl-5,6,7-trihydro-1,2-oxaphosphepine 2-oxide (**41**) only in 18% yield under the same reaction conditions (Scheme 8) [29].

**Scheme 9:**
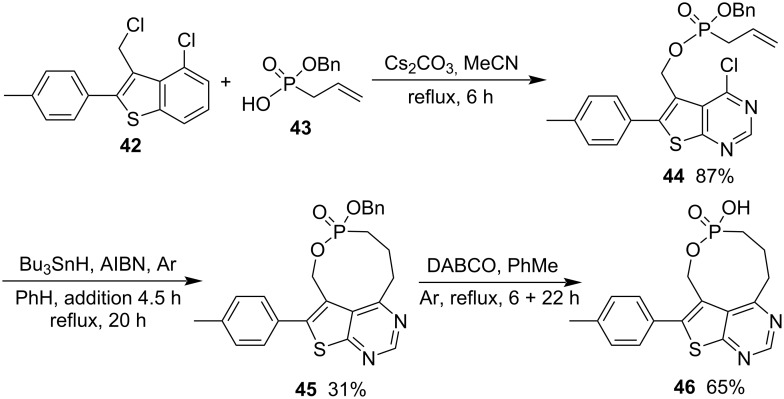
Synthesis of thieno[2,3-*d*]pyrimidine-fused 2-hydroxy-1,2-oxaphosphonane 2-oxide from benzyl hydrogen allylphosphonate and 4-chloro-3-(chloromethyl)-2-(4-methylphenyl)benzo[*b*]thiophene.

To investigate thienopyrimidine-based monophosphonate inhibitors of the human farnesyl pyrophosphate synthase, various thienopyrimidine-derived phosphonate derivatives were synthesized. Benzyl ((4-chloro-6-(4-methylphenyl)thieno[2,3-*d*]pyrimidin-5-yl)methyl) allylphosphonate (**44**) was prepared in 87% yield from 4-chloro-3-(chloromethyl)-2-(4-methylphenyl)benzo[*b*]thiophene (**42**) and benzyl hydrogen allylphosphonate (**43**) via alkylation in the presence of Cs_2_CO_3_ in refluxing acetonitrile for 6 h. It underwent a radical cyclization in refluxing benzene for 20 h to give rise to a nine-membered phostone thieno[2,3-*d*]pyrimidine-fused 2-hydroxy-1,2-oxaphosphonane 2-oxide **46** as a potential inhibitor after the deprotection of the benzyl group in the presence of DABCO in refluxing toluene under argon atmosphere (Scheme 9) [21].

**Scheme 10:**
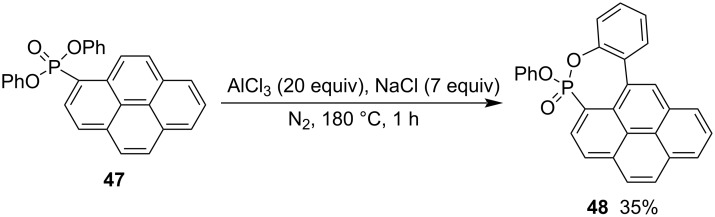
Synthesis of 3-phenoxybenzo[*f*]pyreno[1,10-*cd*][1,2]oxaphosphepine 3-oxide from diphenyl pyren-1-ylphosphonate.

In addition, the intramolecular coupling reaction of diphenyl pyren-1-ylphosphonate (**47**) accomplished the synthesis of 3-phenoxybenzo[*f*]pyreno[1,10-*cd*][1,2]oxaphosphepine 3-oxide (**48**) in 35% yield in the presence of largely excessive amounts of AlCl_3_ and NaCl. Only one example was reported for this class of the synthesis via C–C bond formation (Scheme 10) [30].

#### 1.2 Synthesis via C–O bond formation

**Scheme 11:**
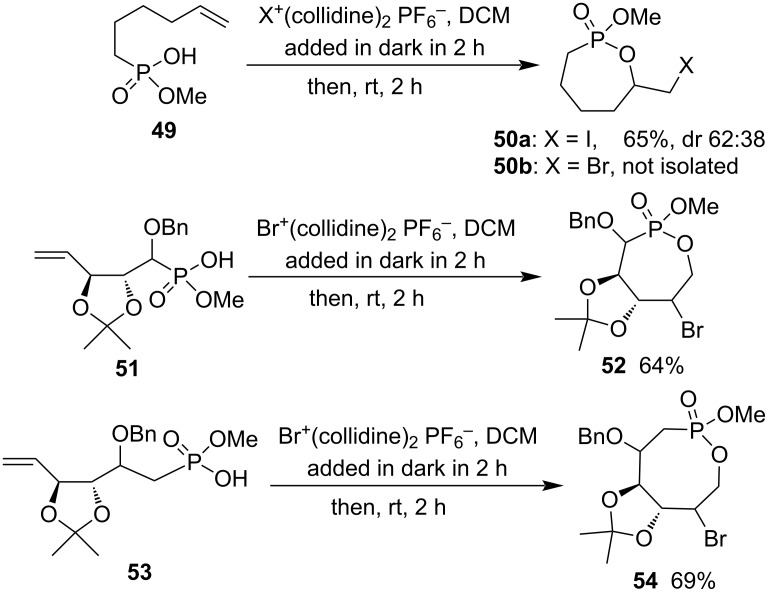
Synthesis of 1,2-oxaphosphepane 2-oxides and 1,2-oxaphosphocane 2-oxide from hydrogen methyl hex-5-en-1-yl/pent-4-enylphosphonates.

Halocyclization has been widely applied in the syntheses of phostone and phostine derivatives via C–O bond formation [13,17]. Both bromo and iodo(biscollidine) hexafluorophosphates were efficient halogenium reagents in the electrophilic halocyclization. Hydrogen methyl hex-5-en-1-ylphosphonate (**49**) generated 2-methoxy-7-iodomethyl-1,2-oxaphosphepane 2-oxide (**50a**) in 65% yield in 62:38 diastereomeric ratio with iodo(biscollidine) hexafluorophosphate as the iodinium agent. However, the corresponding 7-bromomethyl product **50b** was not isolated with bromo(biscollidine) hexafluorophosphate as the brominium reagent. The reactants were further extended to functionalized hydrogen methyl 1,3-dioxolane-fused pent-4-en-1-ylphosphonate **51** with bromo(biscollidine) hexafluorophosphate as the brominium reagent, leading to the corresponding 3-benzyloxy-6-bromo-1,2-oxaphosphepane 2-oxide **52** in 64% yield. The reaction of hydrogen methyl 1,3-dioxolane-fused hex-5-en-1-ylphosphonate **53** with bromo(biscollidine) hexafluorophosphate produced the corresponding 4-benzyloxy-7-bromo-1,2-oxaphosphocane 2-oxide **54** in 69% yield (Scheme 11) [31].

**Scheme 12:**
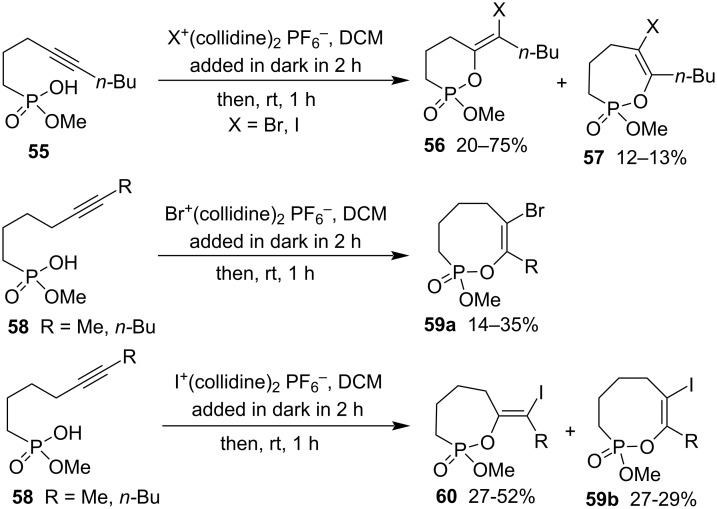
Synthesis of 2-methoxy-1,2-oxaphosphinane 2-oxides, 1,2-oxaphosphepine 2-oxides, 1,2-oxaphosphepane 2-oxides, and 1,2-oxaphosphocine 2-oxides from hydrogen methyl alk-4/5-ynylphosphonates.

When hydrogen methyl non-4-ynylphosphonate (**55**) was employed, the corresponding product 6-(1-halopentylidene)-2-methoxy-1,2-oxaphosphinane 2-oxides **56** were obtained as major products with 7-butyl-6-halo-2-methoxy-3,4,5-trihydro-1,2-oxaphosphepine 2-oxides **57** as byproducts in low yields. The reactants were further extended to hydrogen methyl alk-5-ynylphosphonates **58**, which produced 8-alkyl-7-bromo-2-methoxy-3,4,5,6-tetrahydro-1,2-oxaphosphocine 2-oxides **59a** as sole products in 14–35% yield with bromo(biscollidine) hexafluorophosphate as the reagent, while they generated mixtures of 7-(1-iodoalkylidene)-1,2-oxaphosphepane 2-oxides **60** and 8-alkyl-7-iodo-2-methoxy-3,4,5,6-tetrahydro-1,2-oxaphosphocine 2-oxides **59b** with iodo(biscollidine) hexafluorophosphate as the reagent (Scheme 12) [32].

#### 1.3 Synthesis via P–C bond formation

**Scheme 13:**
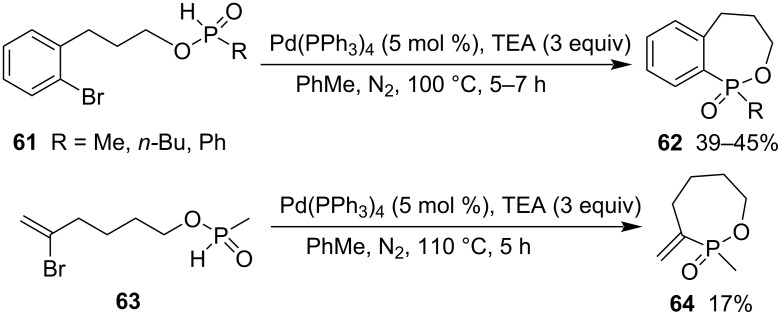
Synthesis of 1,2-azaphosphepane 2-oxide and its benzo derivatives from 5-bromohex-5-en-1-yl methylphosphinate and 3-(2-bromophenyl)propyl alkylphosphinates.

The palladium-catalyzed intramolecular arylation of 3-(2-bromophenyl)propyl alkylphosphinates **61** approached the synthesis of 3,4,5-trihydrobenzo[*c*][1,2]oxaphosphepine 1-oxides **62** in moderate 39–45% yields in the presence of triethylamine in dry toluene at 100 °C [33]. When the substrates were extended to 5-bromohex-5-en-1-yl methylphosphinate (**63**), 2-methyl-3-methylene-1,2-oxaphosphepane 2-oxide (**64**) was obtained in low 17% yield at 110 °C (Scheme 13) [34].

**Scheme 14:**
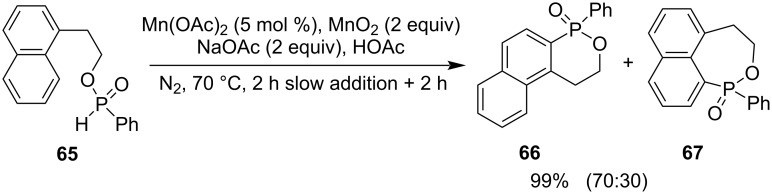
Synthesis of 4-phenyl-1,2-dihydronaphtho[2,1-*c*][1,2]oxaphosphinine 4-oxide and 1-phenyl-3,4-dihydronaphtho[1,8-*cd*][1,2]oxaphosphepine 1-oxide from 2-(naphthalen-1-yl)ethyl phenylphosphinate.

The Mn-catalyzed intramolecular radical arylation of 2-(naphthalen-1-yl)ethyl phenylphosphinate (**65**) gave a mixture of 4-phenyl-1,2-dihydronaphtho[2,1-*c*][1,2]oxaphosphinine 4-oxide (**66**) and 1-phenyl-3,4-dihydronaphtho[1,8-*cd*][1,2]oxaphosphepine 1-oxide (**67**) in 70:30 in a total 99% yield (Scheme 14) [35].

#### 1.4 Synthesis via P–O bond formation

**Scheme 15:**
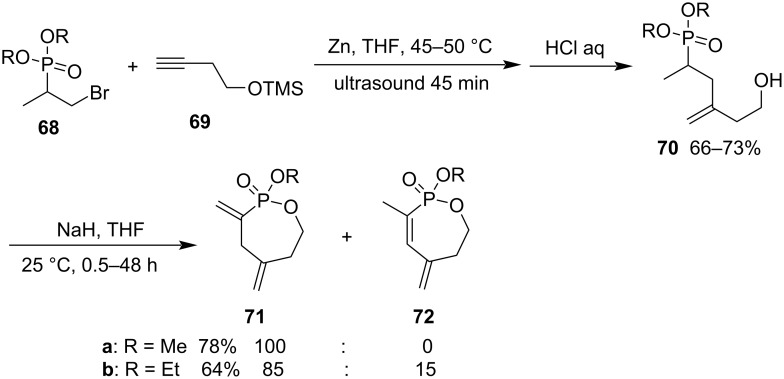
Synthesis of 2-alkoxy-3,5-dimethylene-1,2-oxaphosphepane 2-oxides from dialkyl 2-bromo-1-methylethylphosphonates and trimethylsilyl but-3-ynyl ether.

Intramolecular transesterification is a common method for the synthesis of various phostones and phostines [13,17]. The Zn-catalyzed cross-coupling of dialkyl 2-bromo-1-methylethylphosphonates **68** and trimethylsilyl but-3-ynyl ether (**69**) generated dialkyl 5-hydroxy-1-methyl-3-methylenepentylphosphonates **70** in 66–73% yields under ultrasonic irradiation in THF at 45–50 °C for 45 min followed by treatment with aqueous HCl solution. Further intramolecular transesterificition produced 2-methoxy-3,5-dimethylene-1,2-oxaphosphepane 2-oxide (**71a**) as sole product in 78% yield for dimethyl phosphonate, while the corresponding diethyl phosphonate generated a mixture of 2-ethoxy-3,5-dimethylene-1,2-oxaphosphepane 2-oxide (**71b**) and 2-ethoxy-3-methyl-5-methylene-5,6,7-trihydro-1,2-oxaphosphepine 2-oxide (**72b**) in 85:15 in a total yield of 64% (Scheme 15) [36–37].

**Scheme 16:**
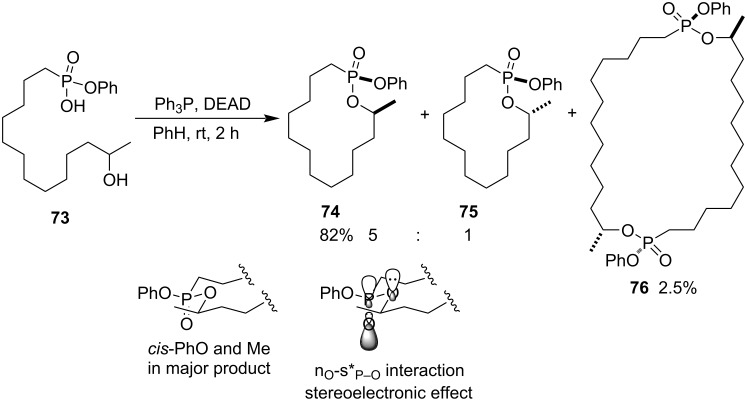
Synthesis of 14-methyl-2-phenoxy-1-oxa-2-phosphacyclotetradecane 2-oxide from phenyl hydrogen (12-hydroxytridecyl)phosphonate.

To prepare a hapten for the production of the catalytic antibody for the catalytic formation of a 14-membered lactone, because it is very difficult for the chemical synthesis of macrocyclic lactones which are the key structural motifs in some biologically active compounds, 14-methyl-2-phenoxy-1-oxa-2-phosphacyclotetradecane 2-oxide (**74**) was synthesized as the hapten from phenyl hydrogen (12-hydroxytridecyl)phosphonate (**73**) via Mitsunobu reaction. The intramolecular esterification gave the cyclized products **74** and **75** in 82% yield with a 5:1 ratio of *anti*/*cis* diastereomers accompanied by a dimeric byproduct **76** in 2.5% yield. The *anti*-diastereomer with *cis*-PhO and Me groups as major product **74** is attributed to the stereoelectronic effect between the n-orbital of the etheric oxygen atom and the antibonding orbital of the P=O bond (the stereoelectronic effect), leading to the more stable *anti*-diastereomer **74** (Scheme 16) [38]. In the phostone **74** was installed a linker 2-(5-aminopentoxy) group via transesterification with benzyl *N*-(5-hydroxypentyl)carbamate followed by hydrogenolysis in the presence of Pd/C as a hapten to produce an antibody. The antibody can catalyze the formation of a difficultly synthesized 14-membered lactone [24].

### 2 Synthesis via annulations

Annulations are alternative strategies for the synthesis of medium and large phostam, phostone, and phostine derivatives through a stepwise bond formation fashion. To date, [5 + 2], [6 + 1], and [7 + 1] annulations have been developed for the synthesis of medium phostams and phostones.

#### 2.1 Synthesis via [5 + 2] annulation

**Scheme 17:**
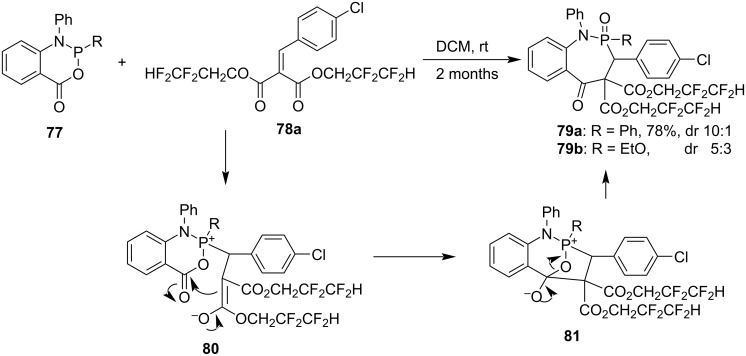
Synthesis of 5-oxo-1,3,5-trihydrobenzo[*f*][1,2]azaphosphepine 2-oxides from 1,2-dihydro-4*H*-benzo[*d*][1,3,2]oxazaphosphinin-4-ones and dialkyl 2-benzylidenemalonate.

A mixture of 2-phenyl/ethoxy-1-phenyl-1,2-dihydro-4*H*-benzo[*d*][1,3,2]oxazaphosphinin-4-ones (**77**) and bis(2,2,3,3-tetrafluoropropyl) 2-(4-chlorobenzylidene)malonate (**78a**) was kept at room temperature for 2 months, giving bis(2,2,3,3-tetrafluoropropyl) 3-(4-chlorophenyl)-2-phenyl/ethoxy-5-oxo-1-phenyl-1,3,5-trihydrobenzo[*f*][1,2]azaphosphepine-4,4-dicarboxylate 2-oxides (**79**) in good yields with moderate to good diastereoselectivities via Michael addition and nucleophilic addition–elimination (Scheme 17) [39]. The synthesis is a [5 + 2] annulation fashion.

**Scheme 18:**
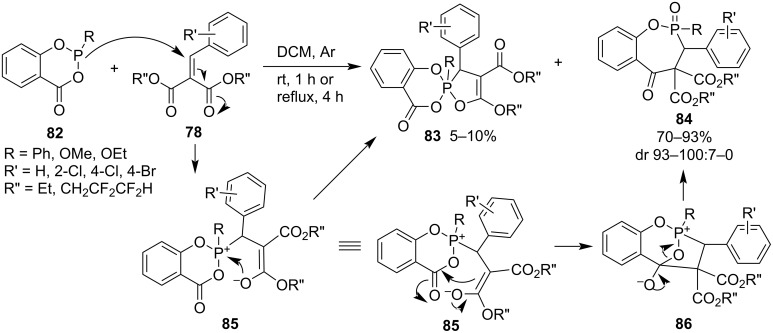
Synthesis of 3-hydrobenzo[*f*][1,2]oxaphosphepin-5(4*H*)-one 2-oxides from 2-phenyl/alkoxy-4*H*-benzo[*d*][1,3,2]dioxaphosphinin-4-ones and dialkyl 2-benzylidenemalonates.

The reactions of 2-phenyl/alkoxy-4*H*-benzo[*d*][1,3,2]dioxaphosphinin-4-ones **82** and dialkyl 2-benzylidenemalonates **78** produced dialkyl 3-aryl-2-phenyl/alkoxy-3-hydrobenzo[*f*][1,2]oxaphosphepin-5(4*H*)-one-4,4-dicarboxylate 2-oxides **84** in good yields with excellent diastereoselectivities accompanied by alkyl 5'-alkoxy-2-phenyl/alkoxy-4-oxo-3'-aryl-2,3'-dihydro-4*H*-2λ^5^-spiro[benzo[*d*][1,3,2]dioxaphosphinine-2,2'-[1,2]oxaphosphole]-4'-carboxylates **83** in 5–10% yield as byproducts. The major products **84** were generated via Michael addition and the nucleophilic addition–elimination of the carbanion of the generated enolate moiety, while the oxyanion of the enolate moiety attacked the phosphorus to form the byproducts **83** (Scheme 18) [40–41].

#### 2.2 Synthesis via [6 + 1] and [7 + 1] annulations

**Scheme 19:**
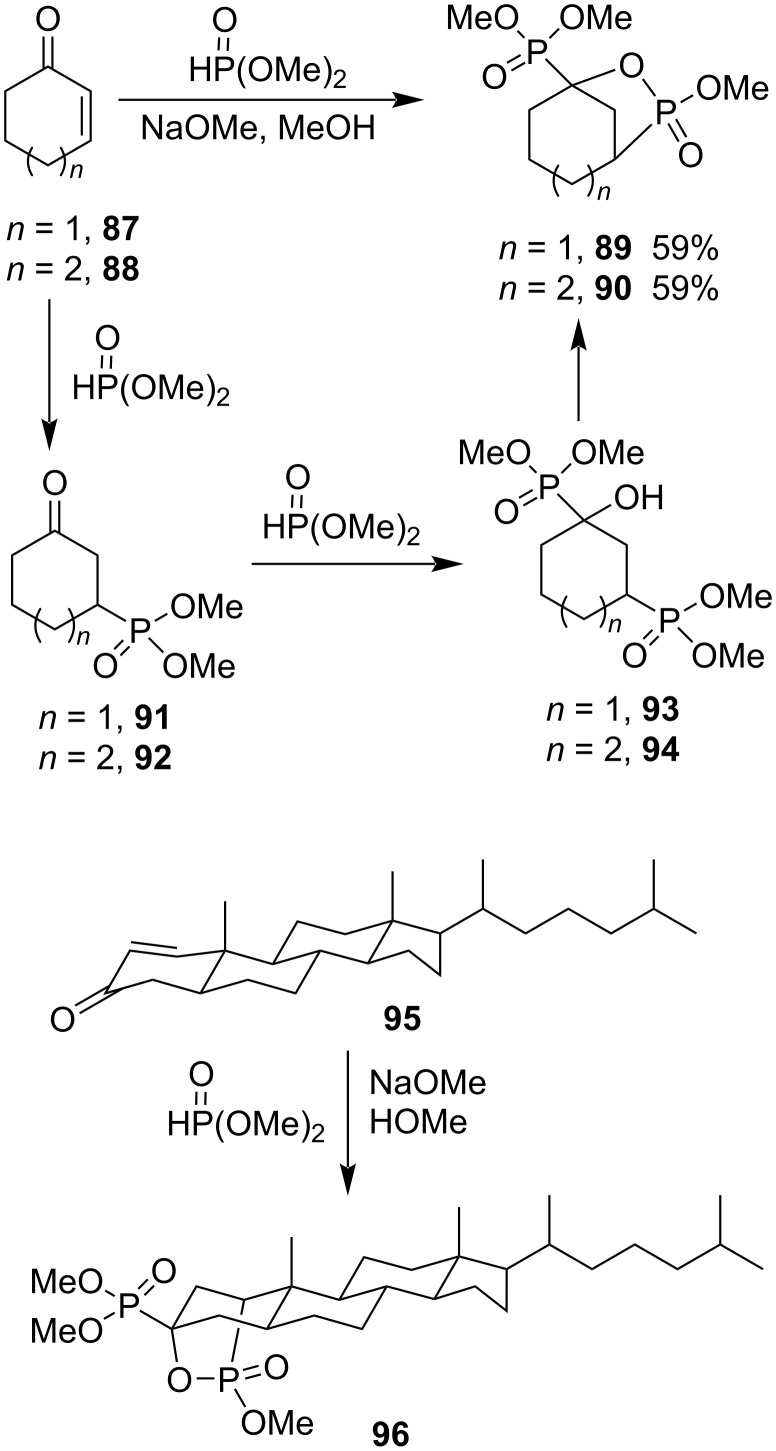
Synthesis of bicyclic seven- and eight-membered phosphotones from cycloalk-2-enones and dimethyl phosphonate.

The reactions of cyclohex-2-enone (**87**) and cyclohept-2-enone (**88**) with dimethyl phosphonate yielded dimethyl bicyclic phostone-phosphonates **89** and **90** under basic conditions in 59% yield via Michael addition, the nucleophilic hydrophosphonylation, and intramolecular transesterification. The method was also applied in the derivatization of a steroid derivative **95**, affording the corresponding product **96** (Scheme 19) [42]. The reactions are [6 + 1] and [7 + 1] annulations for cyclohex-2-enone and cyclohept-2-enone, respectively.

**Scheme 20:**
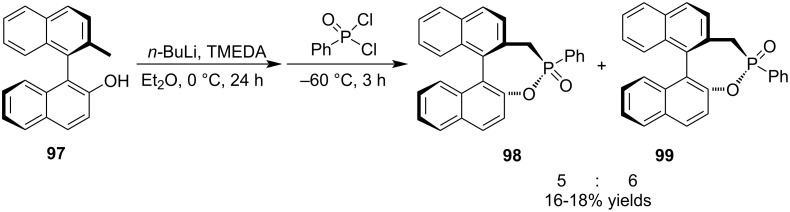
Synthesis of binaphthylene-fused phosphotones from (*M*)-2'-methyl-[1,1'-binaphthalen]-2-ol and phenylphosphonic dichloride.

The treatment of (*M*)-2'-methyl-[1,1'-binaphthalen]-2-ol (**97**) with *n*-BuLi and TMEDA followed by addition of phenylphosphonic dichloride generated a pair of diastereomeric (*M*,4*R*)-4-phenyl-5*H*-dinaphtho[2,1-*d*:1',2'-*f*][1,2]oxaphosphepine 4-oxides **98** and **99** in low yields and low diastereoselectivity via double nucleophilic addition–elimination. Both they are potential chiral phosphorus ligands (Scheme 20) [25].

**Scheme 21:**
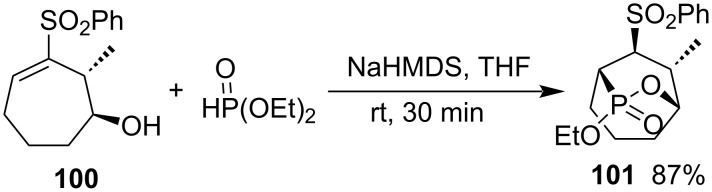
Synthesis of bicyclic phosphotone from (1*S*,2*R*)-2-methyl-3-(phenylsulfonyl)cyclohept-3-en-1-ol and diethyl phosphonate.

When Fuchs and co-workers investigated the conversion of cyclic vinyl sulfones to vinylphosphonates, they found that the reaction of (1*S*,2*R*)-2-methyl-3-(phenylsulfonyl)cyclohept-3-en-1-ol (**100**) and diethyl phosphonate generated (1*R*,5*S*,7*S*,8*S*,9*R*)-7-ethoxy-9-methyl-8-(phenylsulfonyl)-6-oxa-7-phosphabicyclo[3.2.2]nonane 7-oxide (**101**) in 87% yield in the presence of NaHMDS via Michael addition and intramolecular transesterification. The product is a bicycic 1,2-oxaphosphepane 2-oxide derivative (Scheme 21) [43].

## Conclusion

Phostam, phostone, and phostine derivatives are important 1,2-aza/oxaphosphaheterocyclic compounds and phosphorus analogues of lactams and lactones. They show important biological activities. Medium and large phostam, phostone, and phostine derivatives are important biologically active compounds. Several methods have been developed for their synthesis. Their synthetic strategies can be categorized into cyclizations and annulations. The cyclizations have been widely applied for the formation of C–C, C–O, P–C, and P–O bonds in the rings. Annulation include [5 + 2], [6 + 1], and [7 + 1] fashions in the construction of the rings. However, the synthetic methods are still limited, especially for asymmetric synthetic methods. Thus, it is clear that highly stereoselective asymmetric synthetic methods to access various medium and large phostam, phostone, and phostine derivatives are in high demand and should be developed in the near future for potential biological investigations. On the other hand, various methods have been developed for the synthesis of medium and large phostones and phostines, while only limited strategies have been achieved for the preparation of seven- and eight-membered phostams possibly because the P–O bond is more stable than the corresponding P–N bond. Much attention should be paid to the synthesis of different ring size phostams in the future.
